# Supplementary figures and images for: Promising Role of Alkaloids in the Prevention and Treatment of Thyroid Cancer and Autoimmune Thyroid Disease: A Comprehensive Review of the Current Evidence
Source: Int J Mol Sci. 2024 May 15;25(10):5395. doi: 10.3390/ijms25105395 (PMC11121374; doi:10.3390/ijms25105395)

Supplemental Figure S1: Flow chart of the selection of reviewed articles.

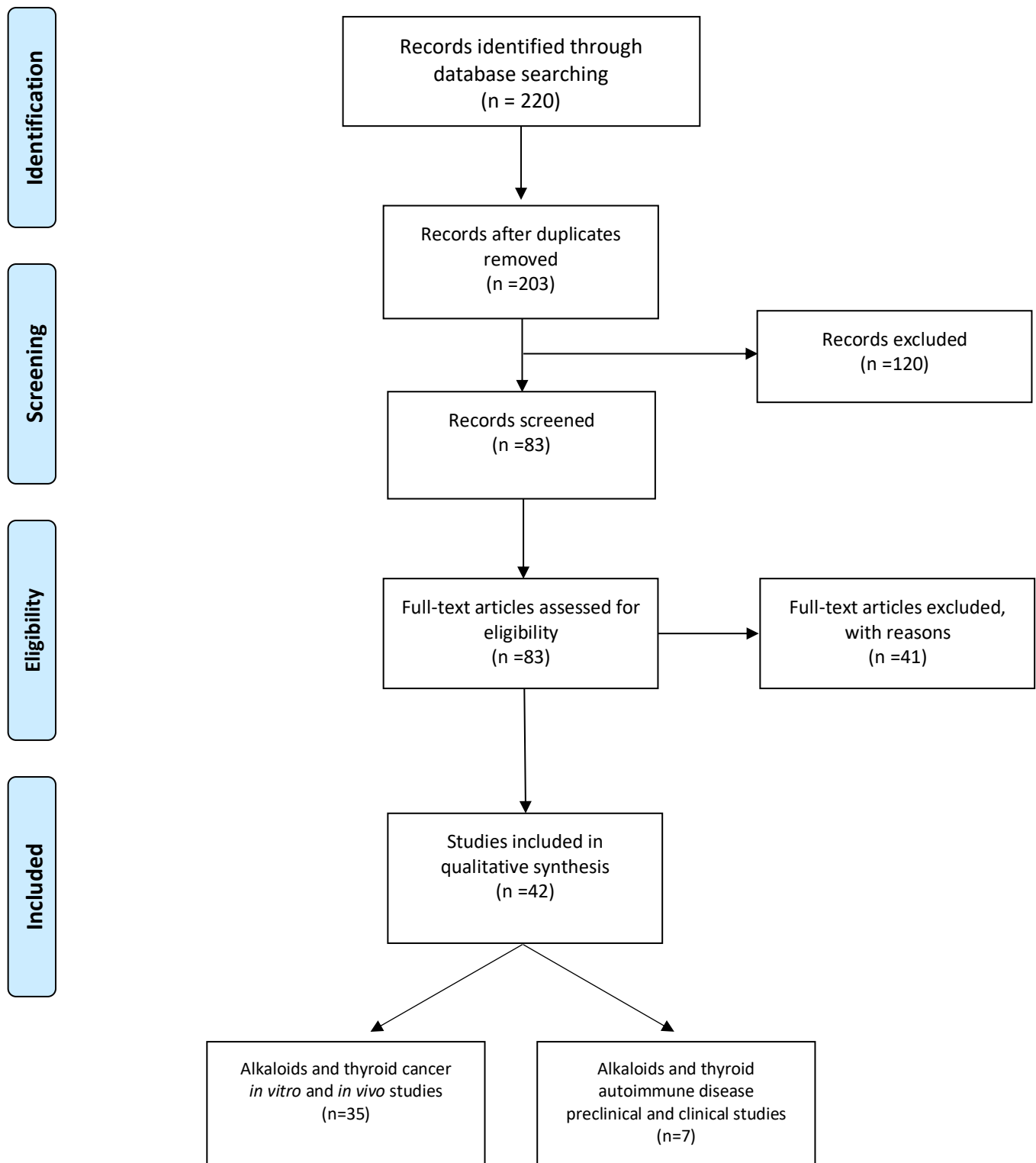

Supplement: Supplementary file 1 [file ijms-25-05395-s001.zip › ijms-2951414-supplementary.pdf]
